# Quantitative proton density mapping with data-driven reception profile correction derived from macromolecular proton fraction

**DOI:** 10.64898/2026.10.13.26365282

**Published:** 2026-10-15

**Authors:** Vasily L. Yarnykh

## Abstract

**Purpose:**

Proton density (PD) and macromolecular proton fraction (MPF) are fundamental MR parameters characterizing water and macromolecular content in tissues. Both parameters can be mapped using a time-efficient MRI protocol, though PD maps require additional corrections for the reception profile (RP) non-uniformity and global scaling. Existing PD mapping methods suffer from uncertainties related to PD normalization to reference tissue. This study aimed to develop a data-driven RP correction method for PD mapping based on MPF as an internal scale.

**Theory and Methods:**

One-parameter model linking PD and MPF was derived under assumption of proportionality between total macromolecular volume and proton concentration. This model with a uniform parameter value across brain tissues generates a surrogate PD map, which is used to estimate RP from an uncorrected PD map. The proposed algorithm was tested against a common PD mapping method utilizing bias-field correction and normalization to cerebrospinal fluid. Brain MRI data were acquired at 3T from seven healthy individuals. The protocol included 3D multi-echo *T*_1_ -, PD-, and magnetization-transfer-weighted gradient-echo sequences. Parametric maps were reconstructed using the single-point MPF mapping algorithm with data-driven *B*_1_ correction.

**Results:**

MPF-based and bias-field corrections yielded similar PD measurements in white and gray matter with no significant bias and remarkably small variability (coefficients of variation <0.6%).

**Conclusions:**

MPF-based RP correction enables computationally simple and robust quantitative PD mapping at no additional cost if MPF maps are available. The main advantage of the proposed method is automatic elimination of global scaling that obviates the need for PD normalization.

## Full Text Availability

The license terms selected by the author(s) for this preprint version do not permit archiving in PMC. The full text is available from the preprint server.

